# When to Extract and When to Conserve Diseased Teeth*Read before the annual meeting of the New Jersey State Dental Association, Atlantic City, New Jersey, July 14 to 16, 1920. Reprinted from *Dental Cosmos*.

**Published:** 1922-02

**Authors:** Thomas B. Hartzell


### WHEN TO EXTRACT AND WHEN TO CONSERVE DISEASED TEETH*

BY THOMAS B. HARTZELL, D.D.S.

The art of mechanical dentistry has been so developed and has reached such a high degree of excellence that it is possible to retain in the jaws of the majority of our patients almost all teeth in relative comfort and efficiency, and the unpleasant suggestion that we must alter our lines of practice and extract teeth from the jaws of our patients which are doing good service as masticating organs is decidedly objectionable to every honest dentist who has practiced conscientious conservation of human teeth.

The question at once arises, Is it necessary to extract the human tooth when the vitality of its pulp has been lost, and, if so, why must we accept this view? To answer that question of why many men now feel that all

* Read before the annual meeting of the New Jersey State Dental Association, Atlantic City, New Jersey, July 14 to 16, 1920. Reprinted from *Dental Cosmos.*

pulpless teeth must be removed from the jaws, it is necessary for me to go back a little and refer to some of the earlier work done in this country by Gilmer and Moody [1] and Henricci and Hartzell [2] on the bacteriology of pulpless teeth and the conclusions arrived at by the foregoing writers, which were, in brief, that devital root ends are to a very large degree the hosts of bacterial growth which produces pathological states in the tissues contiguous to the root ends, and more than that produce pathological conditions in other areas of the body, to which bacteria may travel from the neighborhood of infected root ends. For instance, in the first one hundred and sixty-two pulpless teeth examined by Henrici and Hartzell,[2] one hundred and fifty were found to be the hosts of bacterial growth. These teeth were taken largely from the mouths of hospital patients, and the reason they were removed was to determine, if possible, whether or not these diseased root ends might be in any way responsible for the arthritis, myocarditis and other ailments, because of which the individuals were in the hospital. They were for the most part producing no conscious discomfort to these bedridden patients.

The weighing of this fact, together with the clinical results obtained by the removal of these teeth, tends to lead one to the conclusion that pulpless teeth should all be extracted, particularly when we take into consideration the fact that from many of the cultures produced in this work animals were inoculated, which in turn developed lesions which resembled in many ways the lesions from which the patients were suffering and from which secondary lesions developed in animals. The authors in question were able to obtain cultures of the original type of germ from the inoculated animals.

---

1 Gilmer and Moody: *Journ. Amer. Med. Ass'n*, Dec. 5, 1914, p. 2023.

2 Henrici and Hartzell: *Journ. Amer. Med. Ass'n*, 1915, pp. 1055-1060.

If one were to take the work of Gilmer and Moody, forty cases, or the results of the work by Henrici and Hartzell above referred to, one might fairly conclude that as one hundred and fifty out of one hundred and sixty-two dental foci produced bacterial growth on examination when taken from the jaws, all pulpless teeth should be extracted. As a matter of fact, however, this would be a wrong conclusion for the following reasons:

In the first place, please remember that these earlier studies were made on patients who were already suffering secondary lesions of various types and their resistance to bacterial invasion was low. If we were to extract the same number of pulpless teeth from the jaws of healthy, vigorous individuals, our experience shows that we would find a large number of them bacteria-free and therefore not transplanting infection, so that while the first work was true, without the context of much additional work done upon pulpless teeth properly handled and properly sterilized in the process of treatment and in the mouths of healthy people to begin with, we have not the whole truth, and may arrive at a very different set of conclusions when we have it. Therefore, I sincerely believe that while the earlier articles telling of the damaging results due to metastatic bacterial invasion from pulpless teeth were true, they were not comprehensive enough to tell all of the truth. They only produced a part of the truth, and part truth may oftentimes without the entire truth be made to convey an untruth, and I suggest to my hearers that we can not justly decide as to whether we must remove all pulpless teeth on the evidence of the type produced in the articles referred to because they dealt with sick teeth and sick people altogether. Therefore we must go more deeply into the question and examine many pulpless teeth in the jaws of healthy individuals in order that we may know when to justly destroy valuable masticating mechanisms.

In the meantime we have at hand modern diagnostic methods to determine whether or not patients presenting pulpless teeth are being in any way damaged by these teeth. I therefore recommend the following for a reasonable diagnostic routine with all patients for whom this decision must be made:

There is no reason in the world why dentists should not use the same precautions and the same type of educated judgment in the protection of their patients' best interests as does the physician in the care of his patients or the attorney in the care of his clients. Nothing could be more reasonable than this. Every dentist who assumes to preserve or wreck useful dental mechanisms should either carry out himself or have carried out by competent cooperative procedure by physicians, these complete examinations. When an individual presents pulpless teeth, in which the X-ray betrays no bony disease and in which the evidence of pathological change can not be elicited by palpation of the soft tissues over the root ends or by percussing the teeth themselves with steel instruments, and the patient is otherwise well physically, we are justified in allowing pulpless teeth to remain. In order to justify the extraction of teeth of this character we should have positive evidence that bacterial invasion from them is of sufficient damage to justify their removal.

In order to demonstrate this fact a careful examination of the patient's blood becomes immensely helpful. If by exclusion all other sources of infection are eliminated and we find an increased leukocyte count associated with a secondary anemia, we must conclude that bacterial invasion is going on and that the patient is in danger sufficient to justify extraction.

Still further, the blood examination may reveal a diminished number of leukocytes. For example, a patient presenting a leukocyte count of 6,500 or less and an increased proportion of lymphocytes over phagocytes associated with a secondary anemia, we are given a positive warning that the patient is in serious danger and the extraction should be performed only as rapidly as the patient can bear surgical interference. We are further warned that we should not extract more than a very limited number of teeth at a sitting with safety to the patient, because if the teeth in question or their bony environment are the site of much bacterial growth, the removal of the teeth will of necessity open many blood vessels and convey an immediate shower of bacteria to the blood stream. In the presence of a diminished leukocyte count, the patient will not be able to combat the bacteria suddenly spread in the blood stream. Many patients succumb to an immediate and rapid dissolution under such conditions. However, if such extractions are few in number and the bacterial shower produced well within the patient's ability to destroy, and the extractions are made at intervals sufficiently far apart to permit the destruction of the shower of bacteria, the patient will evidence a steady increase in energy and an overcoming of secondary or metastatic infection which may have arisen from the infected tooth area.

The examination of the urine, while not of such great value to the diagnostician in concluding whether to do a surgical operation and just how much surgery the patient should be compelled to bear at one time, should not be neglected. The presence of casts, albumin, sugar, or red blood cells in the urine indicates grave conditions which must always have a direct bearing on the conservation or destruction of pulpless teeth, particularly if such pulpless teeth present radiographic evidence of deep pyorrhea pockets or granulomatous root ends. If patients present evidence of secondary infection in the general examination and, in addition to that, present a blood picture indicative of a well-fought battle between the bacterium and the leukocyte as evidenced by an increased leukocyte count, we know we should operate and could so operate with greater rapidity than we could if the blood count shows an ill-fought battle between the bacterium and the leukocyte and a decreased leukocyte count. Therefore, I

vigorously recommend that the dental profession of the future follow better diagnostic methods, and when conditions brought out by such methods indicate that direct damage is being wrought through the presence of pulpless teeth, they should be removed. If such evidence is not elicited, we should bear in mind the fact that for fifty years the skill of the dentist has been such that, where conscientious asepsis has been practiced, pulpless teeth have been conserved for thousands of individuals who have died of old age, and the needless removal of good masticating mechanisms when not doing harm is as much to be deprecated as is their retention when doing harm.

